# Subseafloor sulphide deposit formed by pumice replacement mineralisation

**DOI:** 10.1038/s41598-021-87050-z

**Published:** 2021-04-23

**Authors:** Tatsuo Nozaki, Toshiro Nagase, Yutaro Takaya, Toru Yamasaki, Tsubasa Otake, Kotaro Yonezu, Kei Ikehata, Shuhei Totsuka, Kazuya Kitada, Yoshinori Sanada, Yasuhiro Yamada, Jun-ichiro Ishibashi, Hidenori Kumagai, Lena Maeda, the D/V Chikyu Expedition 909 Scientists, Shigeshi Fuchida, Tatsuo Fukuhara, Kei Ikehata, Jun-ichiro Ishibashi, Hirokazu Kato, Masanobu Kawachi, Shinji Kawaguchi, Ryuhei Kawakida, Kazuya Kitada, Shogo Komori, Hiroshi Koshikawa, Kakda Kret, Hidenori Kumagai, Lena Maeda, Yuka Masaki, Yohei Matsui, Iona McIntosh, Kana Minamide, Rena Miyahara, Nobuhiro Mukae, Toshiro Nagase, Shunsuke Nakamura, Tatsuo Nozaki, Masao Ohno, Tsubasa Otake, Masafumi Saitoh, Yoshinori Sanada, Yutaro Takaya, Tomohiro Toki, Junji Torimoto, Shuhei Totsuka, Akihi Tsutsumi, Riki Uehara, Hirotaka Uza, Masayuki Watanabe, Yasuhiro Yamada, Takahiro Yamagishi, Hirofumi Yamamoto, Toru Yamasaki, Kotaro Yonezu

**Affiliations:** 1 Submarine Resources Research Center, Research Institute for Marine Resources Utilization, Japan Agency for Marine-Earth Science and Technology (JAMSTEC), 2-15 Natsushima-cho, Yokosuka, Kanagawa 237-0061 Japan; 2 Frontier Research Center for Energy and Resources, School of Engineering, The University of Tokyo, 7-3-1 Hongo, Bunkyo-ku, Tokyo, 113-8656 Japan; 3 Department of Planetology, Graduate School of Science, Kobe University, 1-1 Rokkodai-cho, Nada-ku, Kobe, Hyogo 657-8501 Japan; 4 Ocean Resources Research Center for Next Generation, Chiba Institute of Technology, 2-17-1 Tsudanuma, Narashino, Chiba 275-0016 Japan; 5 The Tohoku University Museum, The Center for Academic Resources and Archives, Tohoku University, 6-3 Aoba, Aramaki, Aoba-ku, Sendai, Miyagi 980-8578 Japan; 6 Faculty of Science and Engineering, Waseda University, 3-4-1 Okubo, Shinjuku-ku, Tokyo, 169-8555 Japan; 7 Research Institute of Geology and Geoinformation, Geological Survey of Japan (GSJ), National Institute of Advanced Industrial Science and Technology (AIST), Central 7, 1-1-1 Higashi, Tsukuba, Ibaraki 305-8567 Japan; 8 Division of Sustainable Resources Engineering, Faculty of Engineering, Hokkaido University, Kita 13 Nishi 8, Kita-ku, Sapporo, Hokkaido 060-8628 Japan; 9 Department of Earth Resources Engineering, Faculty of Engineering, Kyushu University, 744 Motooka, Nishi-ku, Fukuoka, 819-0395 Japan; 10 Faculty of Life and Environmental Sciences, University of Tsukuba, 1-1-1 Tennodai, Tsukuba, Ibaraki 305-8577 Japan; 11 Department of Earth and Planetary Sciences, Faculty of Science, Kyushu University, 744 Motooka, Nishi-ku, Fukuoka, 819-0395 Japan; 12 Institute for Extra-Cutting-Edge Science and Technology Avant-Garde Research, Japan Agency for Marine-Earth Science and Technology (JAMSTEC), 2-15 Natsushima-cho, Yokosuka, Kanagawa 237-0061 Japan; 13 Institute for Marine-Earth Exploration and Engineering, Japan Agency for Marine-Earth Science and Technology (JAMSTEC), 2-15 Natsushima-cho, Yokosuka, Kanagawa 237-0061 Japan; 14 Graduate School of Integrated Arts and Sciences, Kochi University, 2-5-1 Akebono, Kochi, 780-8520 Japan; 15 Department of Earth Sciences, Royal Holloway University of London, Egham Hill, Surrey, TW20 0EX UK; 16 Center for Regional Environmental Research, National Institute for Environmental Studies, 16-2 Onogawa, Tsukuba, Ibaraki 305-8506 Japan; 17 Project Team for Development of New-Generation Research Protocol for Submarine Resources, Japan Agency for Marine-Earth Science and Technology (JAMSTEC), 2-15 Natsushima-cho, Yokosuka, Kanagawa 237-0061 Japan; 18 Department of Environmental Changes, Faculty of Social and Cultural Studies, Kyushu University, Motooka 744, Nishi-ku, Fukuoka, 819-0395 Japan; 19 Center for Environmental Biology and Ecosystem Studies, National Institute for Environmental Studies, 16-2 Onogawa, Tsukuba, Ibaraki 305-8506 Japan; 20 Research Institute for Geo-Resources and Environment, Geological Survey of Japan (GSJ), National Institute of Advanced Industrial Science and Technology (AIST), Central 7, 1-1-1 Higashi, Tsukuba, Ibaraki 305-8567 Japan; 21 Cosmos Shoji Co., Ltd, 2-11 Kanda Nishiki-cho, Chiyoda-ku, Tokyo, 101-0054 Japan; 22 Volcanoes and Earth’s Interior Research Center, Research Institute for Marine Geodynamics, Japan Agency for Marine-Earth Science and Technology (JAMSTEC), 2-15 Natsushima-cho, Yokosuka, Kanagawa 237-0061 Japan; 23 Department of Urban Management, Graduate School of Engineering, Kyoto University, Nishikyo-ku, Kyoto, 615-8540 Japan; 24 Department of Chemistry, Biology and Marine Science, Faculty of Science, University of the Ryukyus, 1 Senbaru, Nishihara, Okinawa 903-0213 Japan; 25 Department of Physics and Earth Sciences, Faculty of Science, University of the Ryukyus, 1 Senbaru, Nishihara, Okinawa 903-0213 Japan; 26 Research Center for Health and Environmental Risk, National Institute for Environmental Studies, 16-2 Onogawa, Tsukuba, Ibaraki 305-8506 Japan; 27 Present Address: Research Institute for Geo-Resources and Environment, Geological Survey of Japan (GSJ), National Institute of Advanced Industrial Science and Technology (AIST), Central 7, 1-1-1 Higashi, Tsukuba, Ibaraki 305-8567 Japan; 28 Present Address: Faculty of Science and Engineering, Waseda University, 3-4-1 Okubo, Shinjuku-ku, Tokyo, 169-8555 Japan; 29 Present Address: Department of Earth and Planetary Science, Graduate School of Science, The University of Tokyo, 7-3-1 Hongo, Bunkyo-ku, Tokyo, 113-0033 Japan; 30 Present Address: Institut des Sciences de la Terre, Université de Lausanne, Géopolis, 1015 Lausanne, Switzerland

**Keywords:** Ocean sciences, Solid Earth sciences

## Abstract

Seafloor massive sulphide (SMS) deposits, modern analogues of volcanogenic massive sulphide (VMS) deposits on land, represent future resources of base and precious metals. Studies of VMS deposits have proposed two emplacement mechanisms for SMS deposits: exhalative deposition on the seafloor and mineral and void space replacement beneath the seafloor. The details of the latter mechanism are poorly characterised in detail, despite its potentially significant role in global metal cycling throughout Earth’s history, because in-situ studies require costly drilling campaigns to sample SMS deposits. Here, we interpret petrographic, geochemical and geophysical data from drill holes in a modern SMS deposit and demonstrate that it formed via subseafloor replacement of pumice. Samples from the sulphide body and overlying sediment at the Hakurei Site, Izena Hole, middle Okinawa Trough indicate that sulphides initially formed as aggregates of framboidal pyrite and matured into colloform and euhedral pyrite, which were replaced by chalcopyrite, sphalerite and galena. The initial framboidal pyrite is closely associated with altered material derived from pumice, and alternating layers of pumiceous and hemipelagic sediments functioned as a factory of sulphide mineralisation. We infer that anhydrite-rich layers within the hemipelagic sediment forced hydrothermal fluids to flow laterally, controlling precipitation of a sulphide body extending hundreds of meters.

## Introduction

Volcanogenic massive sulphide (VMS) deposits on land are a major source of base and precious metals (Cu + Pb + Zn ± Au ± Ag)^1,2^ and are considered to be fossilised versions of modern seafloor massive sulphide (SMS) deposits. SMS deposits^3,4^, which are under consideration as seafloor mineral resources, form by hydrothermal fluid circulation associated with submarine volcanism^5,6^. Previous research cruises employing dive surveys and subseafloor drilling have yielded basic knowledge of two different emplacement mechanisms of SMS deposits^7–10^, which are (1) exhalative deposition, building chimney and mound structures on the seafloor, and (2) subseafloor replacement of relatively unstable material and volcanic glass by sulphide minerals. VMS deposits on land show evidence of both mechanisms^6,11-13^, but few examples document the entire formation and mineralisation process, including the nascent stage of sulphide formation^12,13^, because subsequent hydrothermal, diagenetic, metamorphic and tectonic activity often has erased any relevant evidence. The subseafloor replacement process is considered to promote the trapping of metals from upwelling hydrothermal fluids and produce large deposits with sizable tonnage^11,13^. Therefore, understanding the formation mechanism of SMS deposits is both scientifically and economically important for its relevance to the ancient records of VMS deposits. However, in-situ research within active SMS deposits by vessels capable of subseafloor drilling, though costly, is the only feasible way to better understand the process of subseafloor sulphide mineralisation in modern SMS deposits.

In 2016, scientific drilling cruise CK16-05 (Expedition 909) of *D/V Chikyu* visited a known subseafloor sulphide body at the Hakurei Site, Izena Hole^14,15^ in the middle Okinawa Trough (Supplementary Figs. S1 and S2). The purposes of this cruise were to (1) investigate the formation mechanism of the sulphide body and (2) determine the physical properties of drill cores to support future development of geophysical survey techniques. We obtained continuous drill cores from the upper part of the subseafloor sulphide body and overlying layers of hemipelagic sediment and pumiceous underwater debris flow deposits, including the boundaries between these materials. Here, we report petrographic, geochemical and geophysical signatures of these drill cores to clarify the formation mechanism of a subseafloor sulphide body via a process we have termed pumice replacement mineralisation.

### Drill hole locations

The Okinawa Trough is a back-arc basin in the East China Sea (Supplementary Fig. S1a). Given its slow estimated extension rate of 3.7 ± 0.06 cm year^–1^ (ref.^16^) and its geomorphic features^17^, the Okinawa Trough is at the nascent stage of basin formation, in the transition from continental rifting to seafloor spreading (Supplementary Fig. S1a). The Izena Hole is a rectangular depression measuring about 6 × 3 km in the southwestward continuation of a chain of Quaternary volcanoes of the Ryukyu Arc in the middle Okinawa Trough^18^ that contains two hydrothermal sites, the JADE Site on the northeastern caldera slope^14,19^ and the Hakurei Site on the southern caldera floor^14,15^ (Supplementary Fig. S1b). We drilled nine holes at the Hakurei Site during cruise CK16-05 (Supplementary Fig. S1c, Supplementary Table S1). Two holes (Holes C9027A and C9027B) were drilled into an exhalative sulphide body called the Northern Mound, four holes (Holes C9025A, C9026A, C9028A and C9032A) penetrated a subseafloor sulphide body without exhalative sulphides other than collapsed chimney and mound sulphides at Hole 9028A (Supplementary Fig. S2), and three holes (Holes C9029A, C9030A and C9031A) were reference sites without sulphide mineralisation either at or below the seafloor. Among the reference sites, cores from Holes C9029A and C9030A are dominated by pumice fragments, whereas turbiditic sediment is dominant at Hole C9031A.

## Results and discussion

### Drill core lithologies

Simplified lithologies and representative scan images of drill cores are shown in Supplementary Figs. S2 and S3, respectively, and depth profiles of constituent mineral abundances are shown in Supplementary Figs. S4–S8 for a set of cores along an E-W transect including the Northern Mound (Holes C9032A, C9025A, C9026A, C9028A and C9027A/B). The shallowest material is an underwater debris flow deposit (pumiceous sediment) consisting of pumice fragments, calcareous foraminifer-rich sediment and hemipelagic sediment (segments 1 and 2 in Supplementary Fig. S3). Its constituent minerals, most of which are primary except for montmorillonite and chlorite, are quartz + muscovite (illite) + albite + calcite + chlorite ± montmorillonite ± pyrite. The underwater debris flow deposit is almost unaltered or weakly altered, but some intervals at Holes C9025A, C9028A and C9032A were moderately altered with or without gas expansion of the drill cores. This deposit is underlain by laminated, unaltered hemipelagic sediment (segment 3 in Supplementary Fig. S3) composed of quartz + muscovite (illite) + albite + chlorite ± pyrite. The base (hanging wall) of the hemipelagic sediment layer is in direct contact with a sulphide body (segments 4, 5, and 7 in Supplementary Fig. S3). The sulphide body consists of a porous material dominated by the sulphide and sulphate minerals pyrite, marcasite, sphalerite/wurtzite, and galena ± barite ± chalcopyrite, and contains two intercalated layers of hemipelagic sediment (segment 6 in Supplementary Fig. S3). The sulphide body is underlain by clay formed by hydrothermal alteration (hereafter, ‘hydrothermally altered clay’) (segments 8–11 in Supplementary Fig. S3). This clay layer is pervasively altered (more than 90% consists of clay minerals); its alteration mineral assemblage comprising muscovite (illite) + chlorite ± K-feldspar is commonly found under conditions of neutral and slightly alkaline hydrothermal alteration^20,21^. This assemblage is consistent with the estimated pH value of 4.7 for the hydrothermal fluid end-member obtained from Izena Hole^22^. About 10–15 m below the sulphide body is a layer of greenish, chlorite-rich, hydrothermally altered clay containing veins of pyrrhotite-cubanite (isocubanite) making up about 5–15 vol% of the material (segment 8 in Supplementary Fig. S3) that can be tracked as a key bed for approximately 200 m between Holes C9026A, C9025A and C9032A.

**Figure 1:**
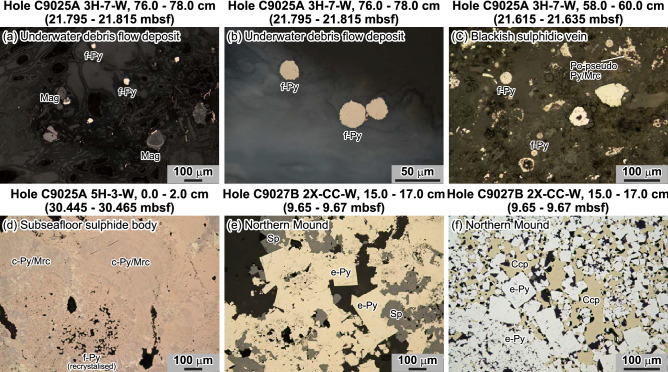
Microphotographs showing pyrite textures under reflected light. All images are in plane-polarised light unless otherwise noted. (**a**,**b**) Framboidal pyrite (f-Py) in pumice fragments of the hanging wall of the underwater debris flow deposit, (**c**) framboidal pyrite with pyrite/marcasite replacing pyrrhotite pseudomorphs (Po-pseudo) in the blackish sulphidic vein of the debris flow deposit, (**d**) colloform pyrite/marcasite (c-Py/Mrc) with recrystallised framboidal pyrite in the upper part of the subseafloor sulphide body (cross-polarised light), (**e**,**f**) euhedral pyrite (e-Py) with sphalerite or chalcopyrite in the sulphide body of the Northern Mound. *Ccp* chalcopyrite, *Mag* magnetite, *Sp* sphalerite.

Pumice fragments in the hanging wall of the underwater debris flow deposit contain numerous aggregates of framboidal pyrite several tens of μm in size (Fig. 1a,b). At Holes C9025A and C9032A, the hanging wall contains blackish sulphidic veins (segment 12 in Supplementary Fig. S3) composed mainly of framboidal pyrite, pseudomorphs of pyrrhotite replaced by pyrite and marcasite, barite and residual plagioclase from the pumice (Fig. 1c). Framboidal pyrite is commonly overgrown by colloform pyrite and marcasite (Fig. 1d), which in turn is overgrown by euhedral pyrite (Fig. 1e,f). Framboidal pyrite is also commonly replaced by other sulphide minerals, such as chalcopyrite, sphalerite and galena, as has been reported at another hydrothermal field in the middle Okinawa Trough^23,24^. This textural evidence suggests a progression of subseafloor sulphide mineralisation that starts with initial framboidal pyrite^13,24–26^. The non-sulphur-bearing gangue minerals also reflect this inferred mineralisation/maturation process: whereas framboidal pyrite is closely associated with amorphous material apparently derived from hydrothermally altered glass from pumice fragments (Supplementary Figs. S9a, S9b, S9d, S9e), the interstices in the later pyrite phases are filled with talc (Supplementary Figs. S9c, S9f.).

### Sulphide-sediment contact zone

**Figure 2:**
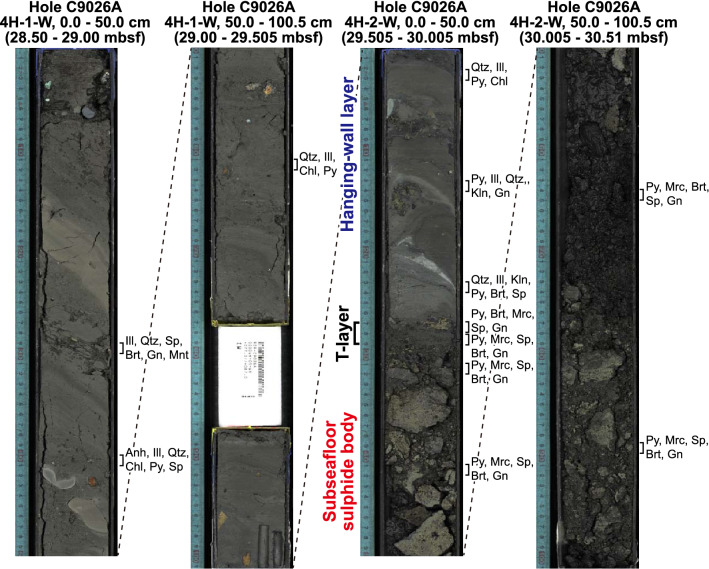
Scanned drill core images showing the contact between hemipelagic sediment and the sulphide body at Hole C9026A. The T-layer, composed of pyrite + barite + marcasite + sphalerite + galena, is 2 cm thick and is interpreted as a cap layer restricting hydrothermal fluid to lateral flow, controlling subseafloor sulphide mineralisation and hydrothermal alteration. *Anh* anhydrite, *Brt* barite, *Chl* chlorite, *Gn* galena, *Ill* illite, *Kln* kaolinite, *Mnt* montmorillonite, *Mrc* marcasite, *Py* pyrite, *Qtz* quartz; Sp, sphalerite.

At Hole C9026A, a 2-cm-thick transitional zone (T-layer hereafter) between the overlying hemipelagic sediment and the sulphide body is composed of pyrite + marcasite + barite + sphalerite + galena (Fig. 2). The T-layer includes the same sulphide and sulphate mineral assemblage seen in the sulphide body, but it also contains a high abundance of barite (up to 3.65 wt% Ba; Supplementary Table S2). The hemipelagic sediment includes two irregular layers 4 and 15 cm above the T-layer containing kaolinite (Fig. 2, Supplementary Fig. S5), a clay mineral that commonly occurs under acidic hydrothermal conditions^20,21^. Its presence is consistent with the estimated end-member pH value of 4.7 for hydrothermal fluid at this site^22^; however, the alteration mineral assemblage of the drill cores indicates hydrothermal alteration under neutral to slightly alkaline conditions. The most plausible explanation for the production of kaolinite in this setting is the separation of hydrothermal fluid into gaseous and liquid phases by boiling^22,27,28^. The selective release of CO_2_ and SO_2_ gases and their ascent into the overlying sediment would produce low pH conditions just above the T-layer. Boiling of hydrothermal fluid is commonly observed at hydrothermal sites in the Okinawa Trough^22,27,28^. As the hanging wall hemipelagic sediment is otherwise almost unaltered, the T-layer may have functioned as a cap layer that confined the hydrothermal fluid to lateral flow, such that only the gas phase leaked into the hanging wall through permeable zones in the T-layer and prompted kaolinite formation.

The localised distribution of kaolinite may be because the T-layer only partially sealed the hydrothermal fluid. Indeed, in Hole C9026A kaolinite occurs both above and beneath the sulphide body (Supplementary Fig. S5), but at Hole C9025A it occurs only beneath the sulphide body (Supplementary Fig. S4). If a layer rich in anhydrite 87 cm above the T-layer (Fig. 2) is taken to be the current cap layer, as discussed below, and if the barite-rich T-layer is taken to be a remnant of a previously existing sulphate-rich cap layer, then the stratigraphic succession of the key minerals barite, kaolinite and anhydrite in ascending order can be explained as a consequence of an anhydrite-rich cap layer advancing into the hanging wall as subseafloor sulphide mineralisation progressed. Anhydrite dissolves after hydrothermal activity ceases as its solubility rises at temperatures less than 300°C^29^. Thus, we interpret the occurrences of barite, kaolinite and anhydrite in ascending order as a record of changes in the position of the cap layer along with overprinting by hydrothermal activity and sulphide mineralisation.

### Geochemical and geophysical characteristics of the lithologies

We present profiles of sulphide mineralisation in the drill cores from five sites in Supplementary Fig. S10 and whole-rock geochemical compositions of all drill cores in Supplementary Table S2. At Holes C9027A and B in the Northern Mound seafloor sulphide body (location in Supplementary Figs. S1 and S2), Cu, Pb and Zn concentrations are 0.02–4.2, 0.03–6.3 and 0.02–12.4 wt% (averaging 0.73, 1.6 and 5.5 wt%, respectively). The Pb and Zn concentrations are enriched in the shallowest few meters compared with deeper parts, and, other than a spike at 4.15 m below the seafloor (mbsf), the Cu concentration is modest near the seafloor and gradually decreases with depth (Supplementary Fig. S10). The Fe concentration gradually increases with depth from 32.9 to 46.1 wt% (Supplementary Table S2), reflecting the higher pyrite abundances at depth (Supplementary Fig. S6). The deepest sample at 63.04 mbsf has the highest Fe and lowest Cu, Pb and Zn concentrations. In our interpretation, primary sphalerite and galena precipitated at depth were remobilised to shallower positions during zone refining when successively hotter hydrothermal fluids came into the system, which is consistent with the higher pyrite and chalcopyrite abundances in the deeper part (Supplementary Fig. S6) and the higher Pb and Zn concentrations in the shallower part (Supplementary Fig. S10).

At Hole C9026A, there are three layers enriched in Cu, Pb and Zn from 30 to 65 mbsf (averaging 0.79, 3.6 and 9.3 wt%, respectively), corresponding to the subseafloor sulphide body (Supplementary Fig. S10). The average Cu concentration is almost equivalent to that of the Northern Mound (0.74 wt%), but the respective Pb and Zn concentrations are 2.3 and 1.7 times higher than those of the Northern Mound (1.6 and 5.5 wt%). These three Cu-Pb–Zn-enriched layers also exist in Hole C9025A at slightly different depths. About 10–15 m below the sulphide body, the layer of hydrothermally altered clay with pyrrhotite-cubanite veins has high Cu concentrations (up to 11.2 wt%). In Hole C9028A, several zones are enriched in Cu, Pb and Zn above the sulphide body and are interpreted to represent collapsed chimneys and mound structures now buried beneath the modern seafloor.

**Figure 3:**
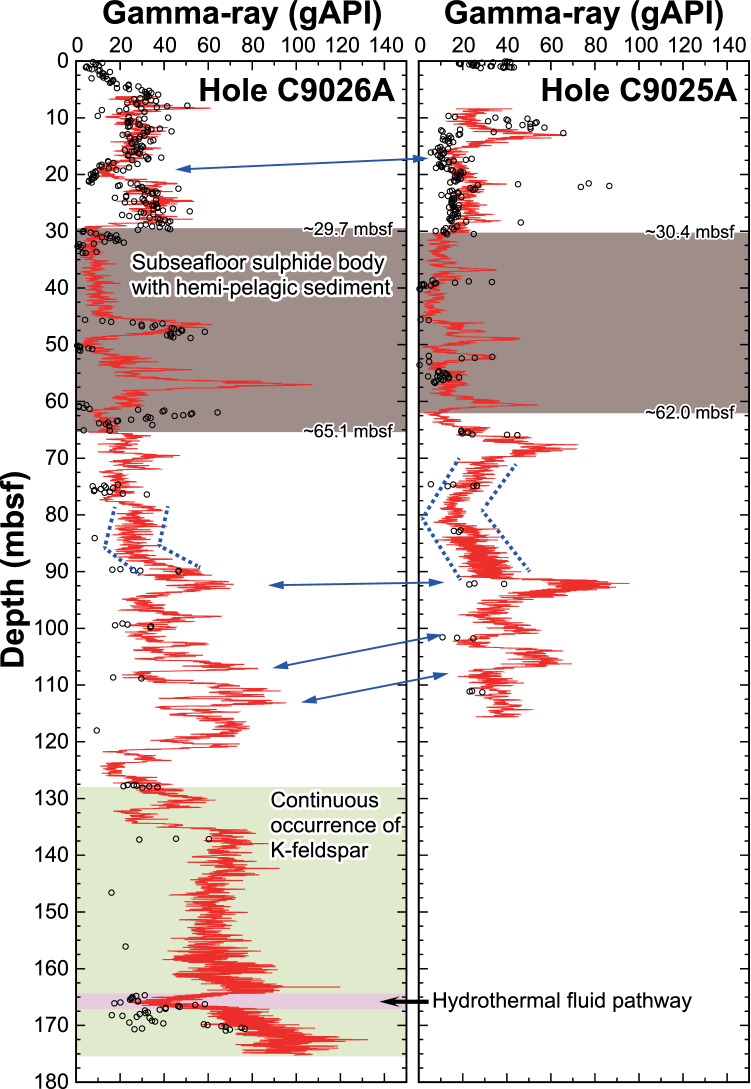
Depth profiles of natural gamma-ray intensity in Holes C9025A and C9026A. Red lines are gamma-ray intensity measured by the PPS71 instrument in the drill holes and open circles are onboard multisensor core logger measurements of recovered drill cores^31^. Areas shaded brown, green and pink represent intervals of the subseafloor sulphide body with intercalated hemipelagic sediment, continuous occurrences of K-feldspar and melting of the inner plastic liner at the high temperatures of the coring operation (interpreted as a hydrothermal fluid channel), respectively. Patterns of the gamma-ray intensities at both holes are quite similar (blue arrows and dotted lines).

Gamma-ray intensity profiles of Holes C9025A and C9026A (Fig. 3) were acquired by through-the-bit logging^30,31^ just after the coring operation to fill gaps in core data at depths where core recovery was poor. In shallow parts of the holes where recovery was good, the gamma-ray profile closely resembles the profile obtained by onboard multisensor core logger measurements of drill cores (Fig. 3), supporting the reliability of the gamma-ray profiling. At greater core depths, the gamma-ray intensities determined by the two methods differ where poor recovery adds up to 9.5 m uncertainty, corresponding to the length of one core barrel, to the subseafloor depth. Natural gamma-ray intensity has a well-known positive correlation with K concentration and is primarily controlled by the occurrence of K-silicate minerals^31^.

The natural gamma-ray intensity profile of Hole C9026A allows the hemipelagic sediment to be distinguished from the underwater debris flow deposit. Positive gamma-ray spikes suggest the presence of at least two layers of hemipelagic sediment within the sulphide body at ca. 48 and 57 mbsf (Fig. 3). The presence of the upper layer was confirmed by the cores, but no core was recovered from the lower layer. The sulphide body and the underlying hydrothermally altered clay from 75 to 85 mbsf are characterised by relatively low gamma-ray intensity. Below 85 mbsf, the profile is controlled by the ratio of illite to chlorite and the occurrence of K-feldspar. High gamma-ray intensity in the deepest part of Hole C9026A is consistent with the continuous occurrence of K-feldspar in the core below 128 mbsf (Supplementary Fig. S5). The gamma-ray profiles of Holes C9026A and C9025A are quite similar, and their stratigraphic profiles can be correlated across the 122 m distance separating the two holes and the 2.5 m difference in water depth, indicating the lateral continuity of the subseafloor strata at the eastern side of the Northern Mound. Both profiles consist, from top to bottom, of underwater debris flow deposits, a subseafloor sulphide body with intercalated hemipelagic sediment, and hydrothermally altered clay having pyrrhotite-cubanite veins in which high gamma-ray intensity peaks appear from 90 to 115 mbsf (Fig. 3). Thus, the gamma-ray logs provide supportive evidence that the subseafloor sulphide body and altered clay, at least from Holes C9025A to C9026A, were formed by the lateral flow of hydrothermal fluid beneath the cap layer of anhydrite-rich clay via mineral and void space replacement in the existing strata. We note that the through-the-bit logging tool utilised in this cruise can be deployed in the borehole immediately after the coring operation and its cost is an order of magnitude lower than that of the logging-while-drilling technique^32^, making it a versatile option for future geophysical logging of seafloor boreholes.

### Process of subseafloor pumice replacement

**Figure 4:**
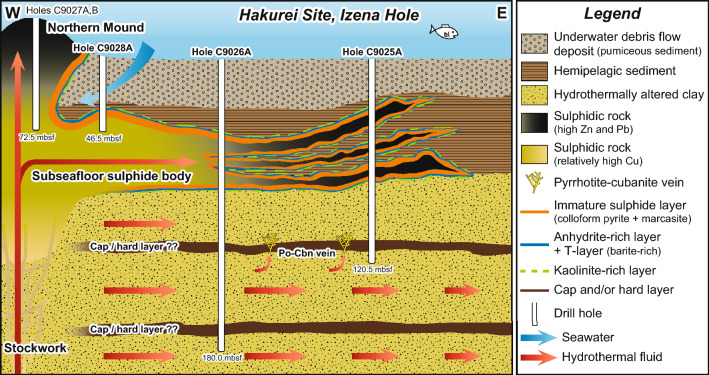
Simplified subseafloor structure of the Hakurei Site, Izena Hole. The subseafloor sulphide body underlies the pumiceous underwater debris flow deposit within hemipelagic sediment and contains at least two hemipelagic sediment layers. The footwall of the sulphide body consists of hydrothermally altered clay with alteration minerals dominated by chlorite + illite ± K-feldspar. A layer of hydrothermally altered clay 10–15 m below the sulphide body contains pyrrhotite-cubanite veins. A deeper cap layer in Hole C9026A appears to confine hot (> 200 °C) hydrothermal fluid. Not to scale. See Supplementary Fig. S11 for more details. *Cbn* cubanite, *Po* pyrrhotite.

Constituent minerals of the subseafloor sulphide body and Northern Mound are pyrite + sphalerite/wurtzite + galena ± chalcopyrite ± marcasite ± pyrrhotite ± barite, an assemblage similar to those of Kuroko-type VMS deposits on land that are interpreted as representing SMS deposits that formed in a back-arc setting^19,33^. However, the subseafloor sulphide body differs as it is porous rather than massive (Fig. 2, Supplementary Fig. S3) and exhibits various textures of sulphide minerals. For example, pyrite, the most abundant sulphide mineral, has framboidal, colloform and euhedral textures in a sequence that tracks sulphide maturation (Fig. 1): framboidal pyrite serves as nuclei for other sulphide minerals^13,24,26^. The depth profiles of sulphide mineral abundance (more abundant pyrite and chalcopyrite in the deeper part) and whole-rock chemical compositions (higher Zn and Pb concentrations in the shallower part) of the Northern Mound are consistent with the formation model reconstructed from Kuroko-type VMS deposits, in which sphalerite and galena are remobilised by hydrothermal overprinting to form zones consisting, in ascending order, of “yellow ore” rich in pyrite and chalcopyrite, “kuroko ore” rich in sphalerite and galena, and barite-rich ore^33^. In contrast, the subseafloor sulphide body to the east has several characteristics that are inconsistent with the conventional formation model. These include (1) the presence of porous rather than massive textures in the subseafloor sulphide body (Fig. 1 and Supplementary Fig. S3), (2) indications of only lateral zone refining (i.e. no clear signs of upward zone refining based on the Cu concentrations in the subseafloor sulphide bodies; Supplementary Fig. S10 and Supplementary Table S2), (3) the presence of nearly unaltered hemipelagic sediment overlying the subseafloor sulphide body and (4) S and Pb isotopic compositions that differ between exhalative mound and subseafloor sulphide body signatures^24,34^. Instead, we propose a model in which mineralisation occurs as subseafloor pumice replacement (Fig. 4).

In our proposed formation model, intercalated layers of hemipelagic sediment and pumice derived from intermittent volcanism function as a reaction space for sulphides, i.e. a sulphide factory (Fig. 4, Supplementary Fig. S11). When hydrothermal fluid enters a permeable pumice layer, anhydrite precipitates in the pores as the hydrothermal fluid reacts with the seawater in the pores. Anhydrite precipitated within the pumice layer or at the contact between pumice and hemipelagic sediment locally reduces the permeability and forms a cap layer that restricts the hydrothermal fluid to lateral flow and raises the temperature beneath it high enough to precipitate sulphide minerals within the pumice deposit. The existence of such an anhydrite cap layer is consistent with observations of a modern SMS deposit hosted in mafic volcaniclastic rocks^35^ and numerical simulations of the seafloor hydrothermal field^36^. This process is similar to the initial formation of chimney structures on the seafloor^33,37^. At this time, hydrothermal alteration of the glass in the pumice layer creates amorphous material as seen in the cores (Supplementary Figs. S9a, S9b, S9d, S9e). Where the vapour phase of boiling hydrothermal fluid can penetrate the anhydrite cap, the pH becomes locally low enough to precipitate kaolinite. After hydrothermal activity ceases and the temperature decreases, the anhydrite dissolves and is replaced by barite and other sulphide minerals, forming the structure we recognise as the T-layer (Fig. 2). Pyrrhotite-cubanite veins in the greenish, chlorite-rich hydrothermally altered clay about 10–15 m below the subseafloor sulphide body are interpreted as having been formed by reducing pristine hydrothermal fluid, with ${f}_{{\mathrm{S}}_{2}}$ and ${f}_{{\mathrm{O}}_{2}}$ low enough to be in the stability field of pyrrhotite precipitation^38^, which was leaked from beneath another cap layer within the hydrothermal fluid reservoir (Fig. 4). Based on onboard visual core descriptions, these pyrrhotite-cubanite veins are isolated from the subseafloor sulphide body by the presence of abundant anhydrite at its base (segment 7 in Supplementary Fig. S3), resulting from reaction with seawater.

Isotopic evidence that Pb is more radiogenic in the subseafloor sulphide body than in the Northern Mound indicates that Pb extraction involves different proportions of hemipelagic sediment and volcanic material in the two localities^34^. Moreover, S isotopic evidence from these cores indicates that framboidal pyrite in pumice fragments of the hanging wall has highly negative δ^34^S values (to –38.9‰) due to bacterial sulphate reduction (BSR), that colloform and euhedral pyrite have δ^34^S values around − 7‰ explained by mixing of magmatic and BSR-derived sulphur^24^, and that galena and sphalerite have positive δ^34^S values (0 to + 3‰) equivalent to magmatic values^15^. As framboidal pyrite is commonly replaced by other sulphide minerals, it is apparent that BSR-derived framboidal pyrite triggers and accelerates subseafloor sulphide mineralisation^24^. The hypothesis that the subseafloor sulphide body was derived from collapsed chimneys and sulphide mounds^39^ cannot explain these geochemical signatures of the subseafloor sulphide body.

The in-place resource represented by the exhalative mound and subseafloor sulphide bodies at the Hakurei Site is estimated to be 7.4 Mt or more^15^, and the mechanism of subseafloor pumice replacement mineralisation presented here, combined with conventional chimney/mound mineralisation^33,37^, creates favourable conditions to form large (high tonnage) SMS deposits and, by inference, large subsequent VMS deposits on land^11,13^. The present study demonstrates the large unexplored potential for subseafloor-replacement-style mineralisation in modern SMS deposits. Comparisons with ancient VMS deposits suggest that the metal resource contained in modern subseafloor strata may be much larger than the resource in exhalative sulphide bodies on the seafloor. Further exploration for these subseafloor deposits requires additional development of geophysical exploration techniques and seafloor drilling.

## Materials and methods

### Samples and onboard drill core handling

Samples used in this study are drill cores obtained by cruise CK16-05 (Expedition 909) of *D/V Chikyu* from 16 November to 15 December 2016 at the Hakurei Site, Izena Hole, in the middle Okinawa Trough^14,15^. The total drilling length of nine holes at eight sites at the Hakurei Site was 834.0 m with a recovered core length of 414.3 m. The vessel’s hydraulic piston coring system (HPCS^40^: https://www.jamstec.go.jp/chikyu/e/about/drilling/coring.html) and short-advance HPCS (SHPCS) were used, achieving core recovery exceeding 80% until hard layers required the use of the extended shoe coring system (ESCS^40^), and subsequent recovery was so poor that the total average core recovery was 49.7%. Although cruise CK16-05 was a domestic cruise by JAMSTEC, drill cores were treated according to an International Ocean Discovery Program (IODP) protocol. We conducted the following sampling and measurements on the cores: (1) head space gas sampling for CH_4_ and H_2_ measurements, (2) X-ray computed tomography measurements, (3) multisensor core logger measurements, (4) scanned images of the split drill cores, (5) visual core description with thin section observations, (6) squeezing interstitial water for salinity, pH, alkalinity, Cl, NH_4_, H_2_S, Si and NO_3_ measurements, (7) physical property measurements such as moisture, density, P-wave velocity, formation factor, penetrometer hardness, thermal conductivity, radioactivity, impedance and rough geochemical compositions by portable-X-ray fluorescence, (8) X-ray diffraction (XRD) analysis, (9) scanning electron microscope observation and (10) microbial incubation. Except for the visual core description and XRD analysis, which were conducted onboard, all microscopic and geochemical results were obtained from shore-based observations and analyses. Detailed information on each drill hole is given in Supplementary Table S1.

### X-ray diffraction analysis

In total, 521 samples were prepared for onboard XRD analyses. Samples were selected to represent all typical lithologies. Around 5 cm^3^ of sample was dried in a vacuum dryer for more than 24 h, followed by milling in a multi-bead shocker (Yasui Kikai Co., PV1103). Samples larger than 2 cm^3^ were pulverised in a tungsten carbide mortar. Powder XRD analyses were performed by a PANalytical CubiX PRO (PW3800) diffractometer equipped with a Cu source, generator voltage of 45 kV and current of 40 mA. The XRD operating conditions were set to step scans from 2° to 60° 2 θ in 5800 steps with step spacing of 0.01° and scan step time of 0.1 s. Diffraction data were analysed using the manufacturer’s diffraction evaluation software (X’Pert HighScore Version 2.1) combined with a crystal database from the International Centre for Diffraction Data. XRD results at Holes C9025A, C9026A, C9027A/B, C9028A and C9032A are given in Supplementary Figs. S4–S8 as well as an approximate mode of occurrence of each constituent mineral, as determined by the Rietveld method using the highest intensity values in the strongest peak of each constituent mineral. Because peak heights of each constituent mineral may be influenced by factors other than abundance (such as crystallinity), these results are qualitative and abundances of clay and phyllosilicate minerals, in particular, were likely to be underestimated.

### Scanning electron microscope observations

Crystallographic observations of drill cores were conducted by a field-emission scanning electron microscope (JEOL JSM-7001F) at Tohoku University with an accelerating voltage of 15 kV and current of 1.41 nA. The drill cores were cut with a diamond saw and polished by successive diamond pastes of 3 and 1 μm with mechanical oil. The samples were then coated with carbon to minimise electron charging.

### Inductively coupled plasma quadrupole mass spectrometry

Major and trace element analyses were performed by an inductively coupled plasma quadrupole mass spectrometer (ICP-QMS; Agilent 7500ce) at JAMSTEC (Supplementary Table S2). In total, 456 subsamples of drill cores from Holes C9025A, C9026A, C9027A/B, C9028A and C9032A were cut with a diamond saw and pulverised in an agate mortar and pestle. Powdered samples weighing ca. 50 mg were dissolved by HNO_3_-HClO_4_-HF digestion in Teflon PFA screw-cap beakers, then heated overnight on a hot plate at 110 °C. The digested samples were progressively evaporated at 110 °C for more than 12 h, 130 °C for 3 h and 160 °C until dryness. The residue was dissolved in 5 mL Milli-Q deionised water combined with 4 mL HNO_3_ and 1 mL HCl, then further diluted to 1:100 by mass (total dilution factor ca. 20,000) before introduction into the ICP-QMS. Details of these analytical procedures, including drift and interference correction methods, were reported in refs. ^41,42^.

### Geophysical downhole through-the-bit logging

A PPS71 geothermal logging tool (Pioneer Petrotech Services Inc.) was used in cruise CK16-05 (Exp. 909). This tool is designed for high-temperature downhole conditions in terrestrial geothermal wells and is also suitable for seafloor hydrothermal sites. Geophysical Survey Co., Ltd and JAMSTEC made further improvements for high-temperature through-the-bit logging including a battery protector, protection chamber, vibration prevention with centraliser and operation methods to safely release and retrieve tools by adding a link jar and sinker bar^31^. The tool consists of a casing collar locator, gamma-ray detector, resistance temperature detector (RTD) and pressure sensor. The robust electronics housed in a vacuum flask allow this tool to perform measurements at 350 °C for 4 h. The sensitivity of gamma-ray measurements is 1.165 cps gAPI^–1^. The PPS71 is equipped with a Pt1000 four-wire RTD sensor for precise temperature measurement with an accuracy of ± 0.5 °C. A silicon-sapphire pressure sensor is used for pressure measurements with an accuracy of ± 0.03% full scale (10,000 psi). These data are stored in the built-in memory. After the coring operation, the drill bit was pulled up at a rate of 0.6–3 m min^–1^ to at least 5 m above the top of the target zone and the PPS71 was installed through the drill bit using a wire line. The PPS71 monitored gamma-ray, temperature and pressure within the drill hole at a sampling rate of 10 Hz. Attenuation of gamma-ray intensity due to the protection chamber was corrected by a prior calibration test in a petroleum drilling well on land with and without the protection chamber. Detailed configuration, data handling and operation of the PPS71 was described in refs.^31,43^.

## Supplementary Information

Supplementary Figures.

Supplementary Table S1.

Supplementary Table S2.
