# Supplementary material for: Subseafloor sulphide deposit formed by pumice replacement mineralisation
Source: Sci Rep. 2021 Apr 23;11:8809. doi: 10.1038/s41598-021-87050-z (PMC8065033; doi:10.1038/s41598-021-87050-z)
Supplement: Supplementary file 1 — Supplementary Figures. [file 41598_2021_87050_MOESM1_ESM.pdf]

Subseafloor sulphide deposit formed by pumice replacement mineralisation

Tatsuo Nozaki[1,2,3,4]✉, Toshiro Nagase[5], Yutaro Takaya[6,1,4], Toru Yamasaki[7], Tsubasa Otake[8], Kotaro Yonezu[9], Kei Ikehata[10], Shuhei Totsuka[11,†], Kazuya Kitada[12], Yoshinori Sanada[13], Yasuhiro Yamada[13,14,15], Jun-ichiro Ishibashi[11], Hidenori Kumagai[1], Lena Maeda[13] & the D/V Chikyu Expedition 909 Scientists

[1]Submarine Resources Research Center, Research Institute for Marine Resources Utilization, Japan Agency for Marine-Earth Science and Technology (JAMSTEC), 2-15 Natsushima-cho, Yokosuka, Kanagawa 237-0061, Japan

[2]Frontier Research Center for Energy and Resources, School of Engineering, The University of Tokyo, 7-3-1 Hongo, Bunkyo-ku, Tokyo 113-8656, Japan

[3]Department of Planetology, Graduate School of Science, Kobe University, 1-1 Rokkodai-cho, Nada-ku, Kobe, Hyogo 657-8501, Japan

[4]Ocean Resources Research Center for Next Generation, Chiba Institute of Technology, 2-17-1 Tsudanuma, Narashino, Chiba 275-0016, Japan

[5]The Tohoku University Museum, The Center for Academic Resources and Archives,

Tohoku University, 6-3 Aoba, Aramaki, Aoba-ku, Sendai, Miyagi 980-8578, Japan

[6]Faculty of Science and Engineering, Waseda University, 3-4-1 Okubo, Shinjuku-ku, Tokyo 169-8555, Japan

[7]Research Institute of Geology and Geoinformation, Geological Survey of Japan (GSJ), National Institute of Advanced Industrial Science and Technology (AIST), Central 7, 1-1-1 Higashi, Tsukuba, Ibaraki 305-8567, Japan

[8]Division of Sustainable Resources Engineering, Faculty of Engineering, Hokkaido University, Kita 13 Nishi 8, Kita-ku, Sapporo, Hokkaido 060-8628, Japan

[9]Department of Earth Resources Engineering, Faculty of Engineering, Kyushu University, 744 Motooka, Nishi-ku, Fukuoka 819-0395, Japan

[10]Faculty of Life and Environmental Sciences, University of Tsukuba, 1-1-1 Tennodai, Tsukuba, Ibaraki 305-8577, Japan

[11]Department of Earth and Planetary Sciences, Faculty of Science, Kyushu University, 744 Motooka, Nishi-ku, Fukuoka 819-0395, Japan

[12]Institute for Extra-cutting-edge Science and Technology Avant-garde Research, Japan Agency for Marine-Earth Science and Technology (JAMSTEC), 2-15 Natsushima-cho, Yokosuka, Kanagawa 237-0061, Japan

[13]Institute for Marine-Earth Exploration and Engineering, Japan Agency for

Marine-Earth Science and Technology (JAMSTEC), 2-15 Natsushima-cho, Yokosuka, Kanagawa 237-0061, Japan

[14]Graduate School of Integrated Arts and Sciences, Kochi University, 2-5-1 Akebono, Kochi 780-8520, Japan

[15]Department of Earth Sciences, Royal Holloway University of London, Egham Hill, Surrey TW20 0EX, United Kingdom

[†]Present address: Research Institute for Geo-Resources and Environment, Geological Survey of Japan (GSJ), National Institute of Advanced Industrial Science and Technology (AIST), Central 7, 1-1-1 Higashi, Tsukuba, Ibaraki 305–8567, Japan

✉e-mail:nozaki@jamstec.go.jp

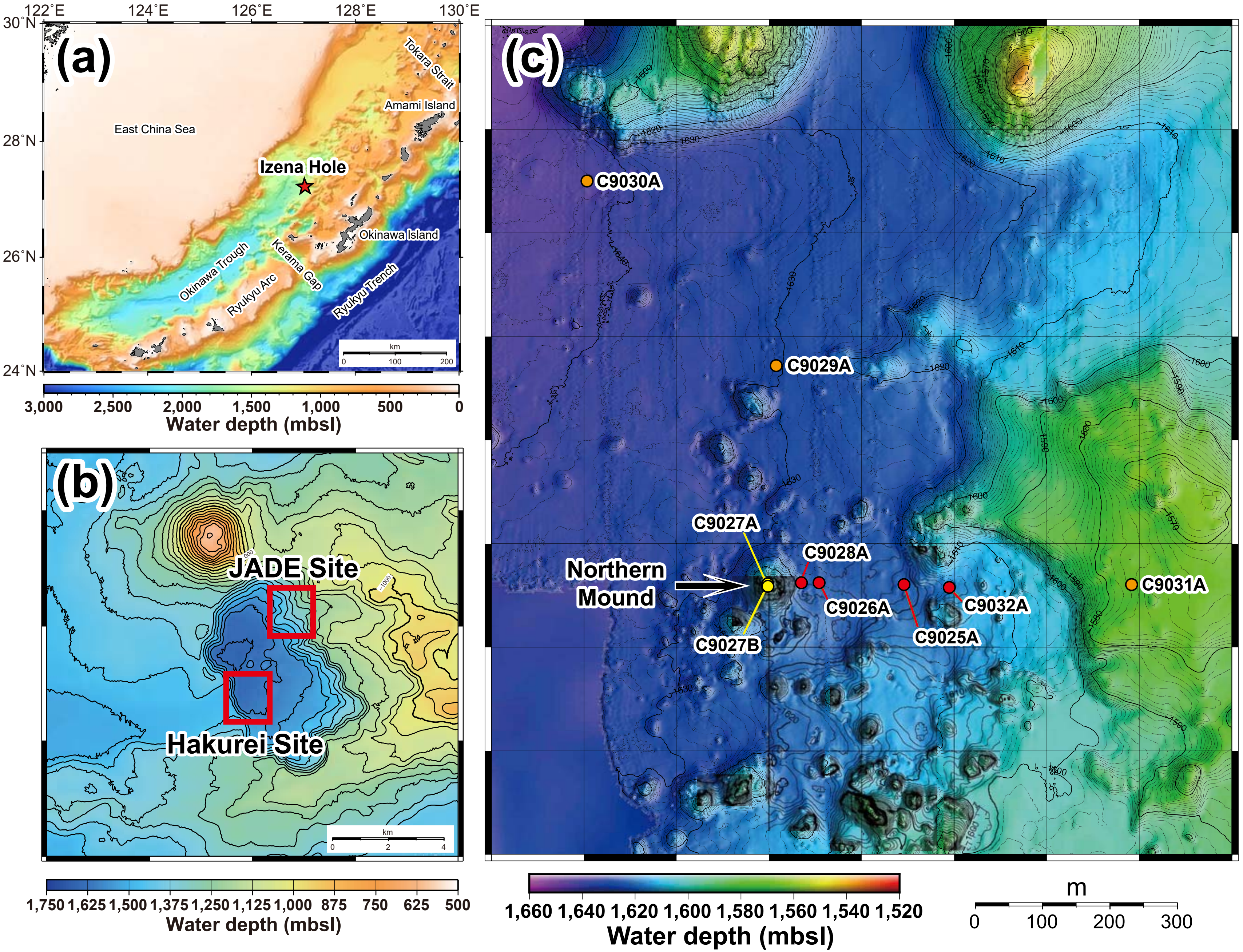

Supplementary Fig. S1 | Location and bathymetry of the Hakurei Site, Izena Hole, middle Okinawa Trough. (**a**) Location of Izena Hole, (**b**) bathymetric map of the Hakurei and JADE hydrothermal sites in Izena Hole[14] and (**c**) detailed (2 m contour) bathymetric map of the Hakurei Site and drill hole locations. Colours of the circles indicate holes encountering the subseafloor sulphide body (red), Northern Mound (yellow) and reference sites without any sulphide mineralisation (orange). Bathymetric data of (**a**) and (**b**) are from the global dataset ETOPO1[44] and data of (**c**) were obtained during cruise YK14-17 (http://www.godac.jamstec.go.jp/darwin/cruise/yokosuka/yk14-17/e) of *R/V Yokosuka* using a SEABAT7125 multi-beam echo sounder mounted on *AUV Urashima* (sounding frequency 400 kHz)[45]. These maps were drawn using a freesoftwere drawn using a freeware software (Generic Mapping Tools Version6)[46].

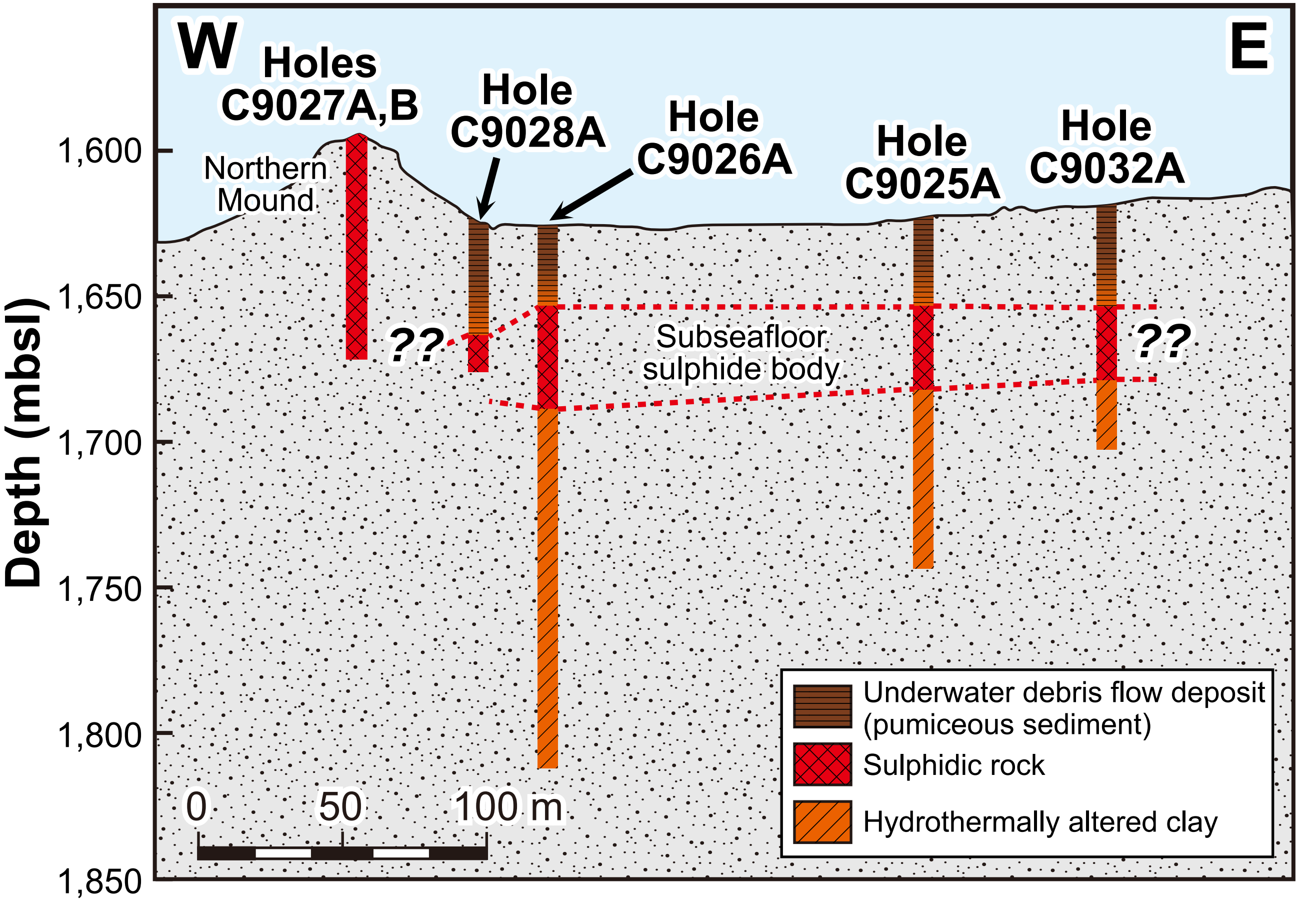

Supplementary Fig. S2 | Simplified lithology of six drill holes along an E-W transect including the Northern Mound. The base of the underwater debris flow deposit is composed of almost unaltered hemipelagic sediment. At least two hemipelagic sediment layers are intercalated in the subseafloor sulphide body at Holes C9026A and C9025A. The uppermost part of the footwall hydrothermally altered clay includes pyrrhotite-cubanite (isocubanite) veins that can be correlated in Holes C9026A, C9025A and C9028A as a key bed. Drill cores sampling the area between the Northern Mound and subseafloor sulphide body or the eastward extension of the subseafloor sulphide body beyond Hole C9032A were not obtained in this cruise.

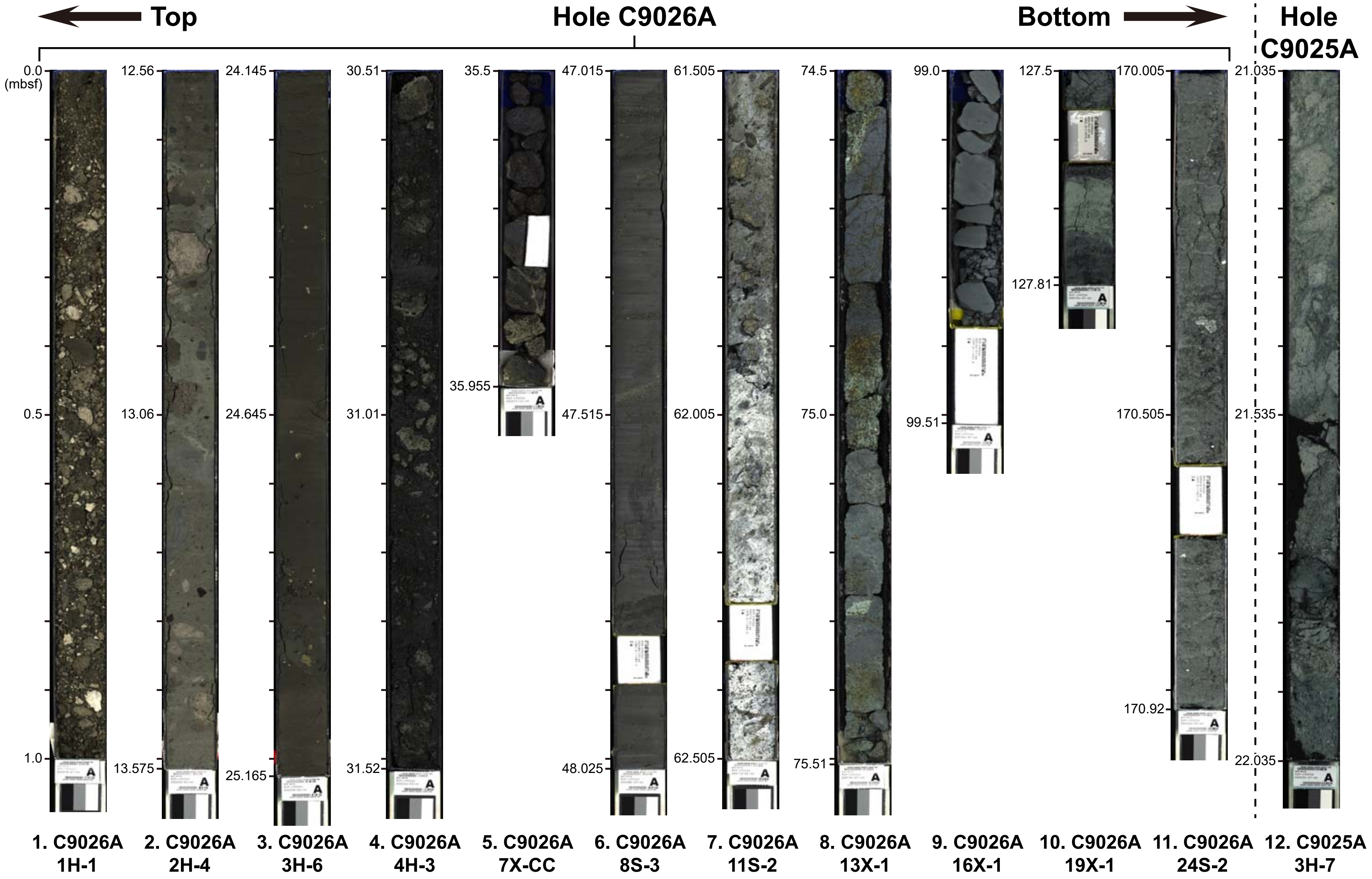

Supplementary Fig. S3 | Representative scanned drill core images covering typical lithologies of Hole C9026A. From top to bottom, the drill cores are composed of underwater debris flow deposit (pumiceous sediment) (1, 2), hanging wall hemi-pelagic sediment (3), subseafloor sulphide body (4, 5, 7) with intercalated hemipelagic sediment (6), hydrothermally altered clay with pyrrhotite-cubanite (isocubanite) veins (8) and hydrothermally altered clay containing muscovite (illite) + chlorite ± K-feldspar (9–11). Blackish sulphidic vein in debris-flow material in Hole C9025A is also shown (12); this material also occurs in Hole C9032A.

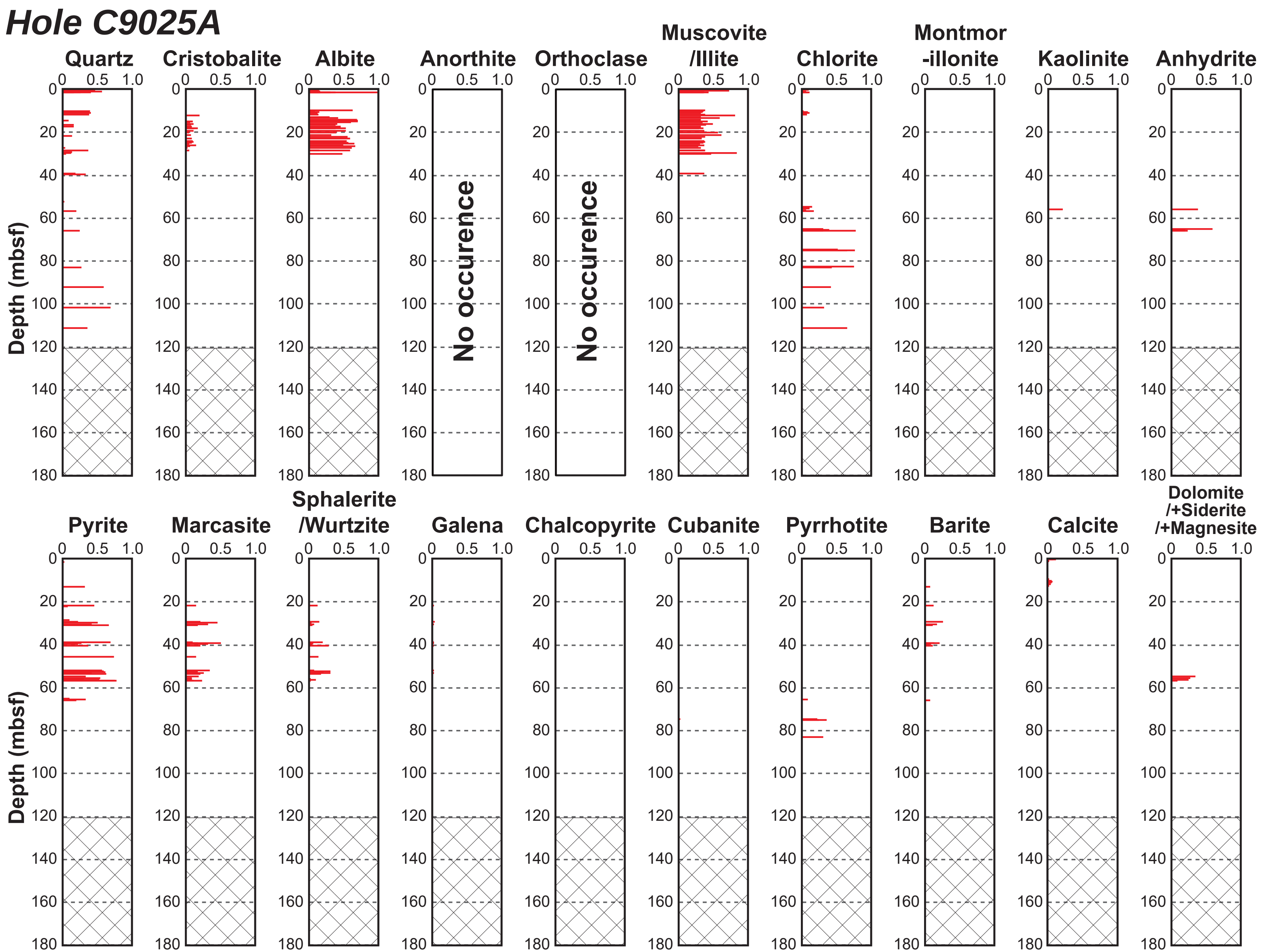

Supplementary Fig. S4 | Depth profile of constituent mineral fractions in Hole C9025A determined by onboard XRD analysis. Red bars indicate mineral fractions, determined by the Rietveld method using the highest intensity values in the strongest peak for each mineral. Because peak heights may be influenced by factors other than abundance, these results are qualitative, and clay and phyllosilicate minerals are especially likely to be underestimated. Hatched intervals represent depths not drilled.

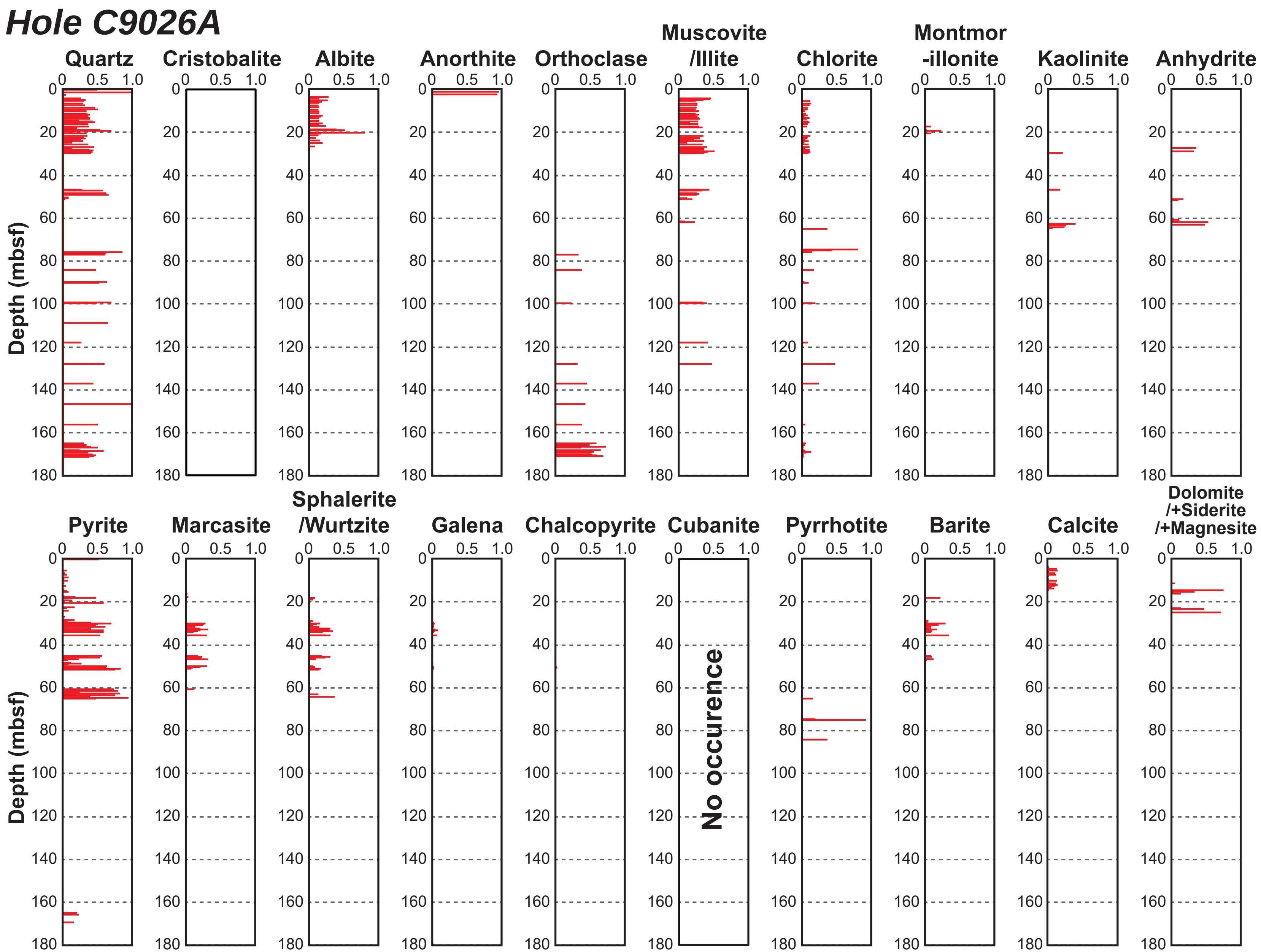

Supplementary Fig. S5 | Depth profile of constituent mineral fractions in Hole C9026A. Details are the same as in Supplementary Fig. S4.

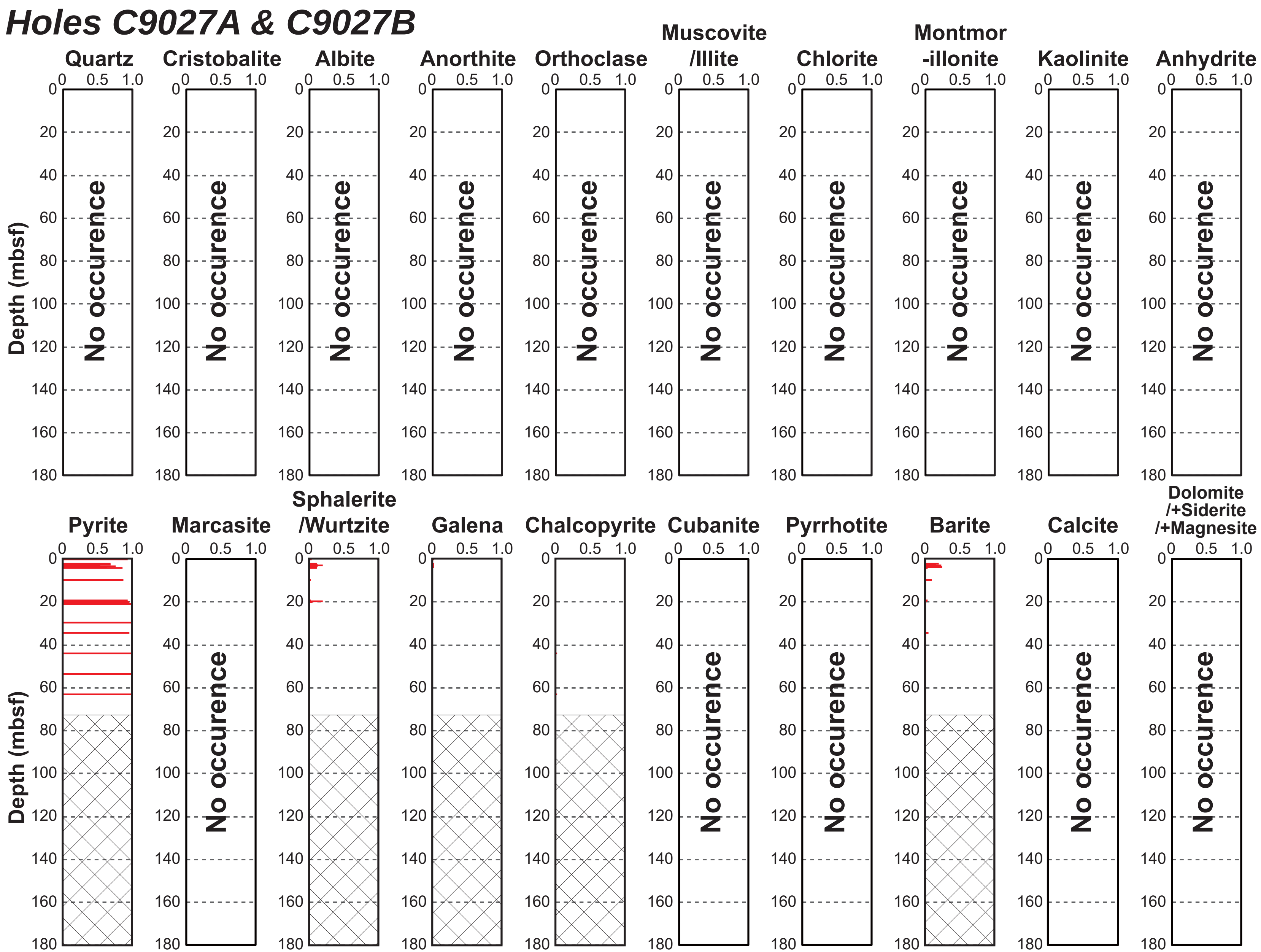

Supplementary Fig. S6 | Depth profile of constituent mineral fractions in Holes C9027A and C9027B. Details are the same as in Supplementary Fig. S4.

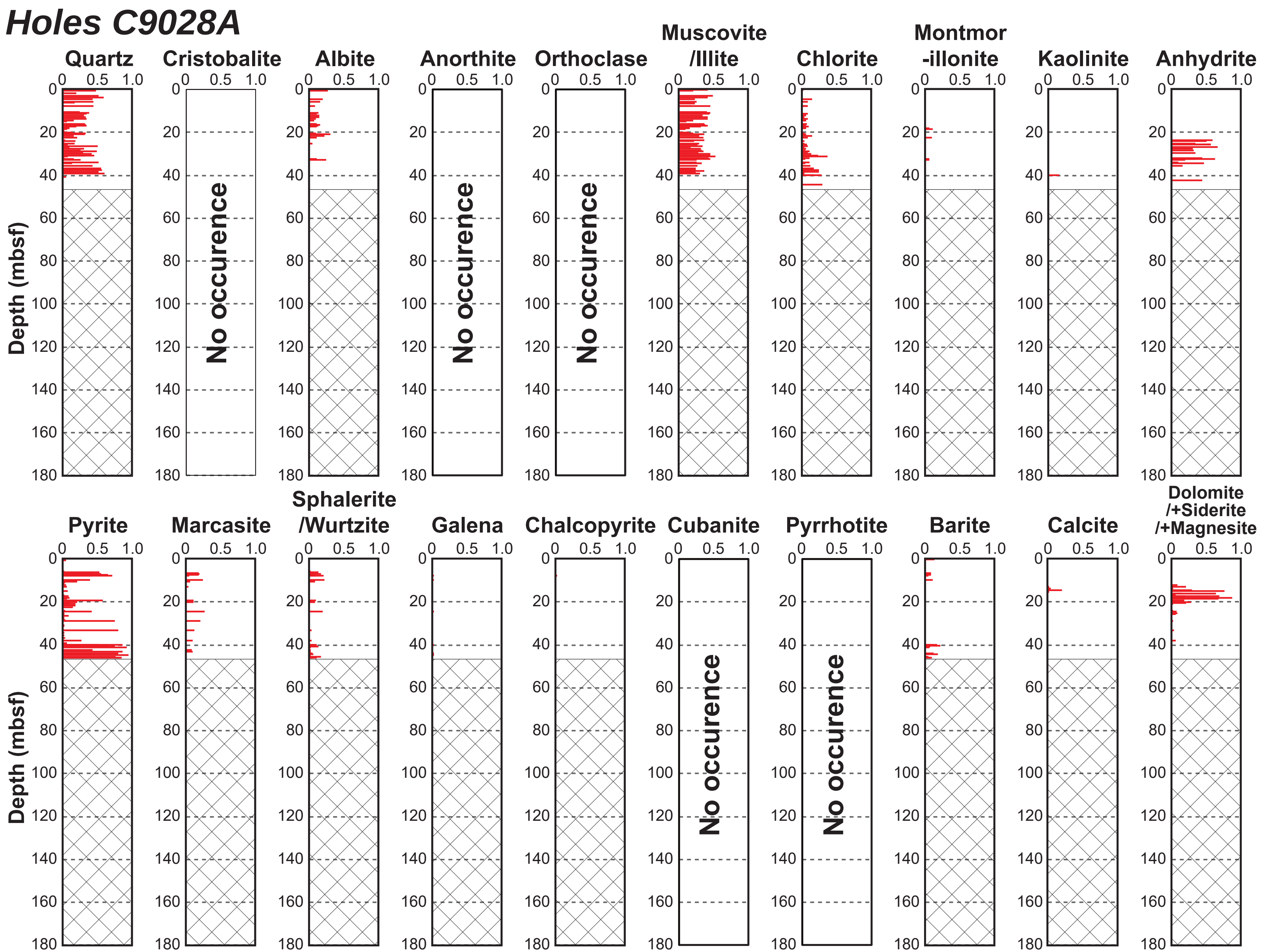

Supplementary Fig. S7 | Depth profile of constituent mineral fractions in Hole C9028A. Details are the same as in Supplementary Fig. S4.

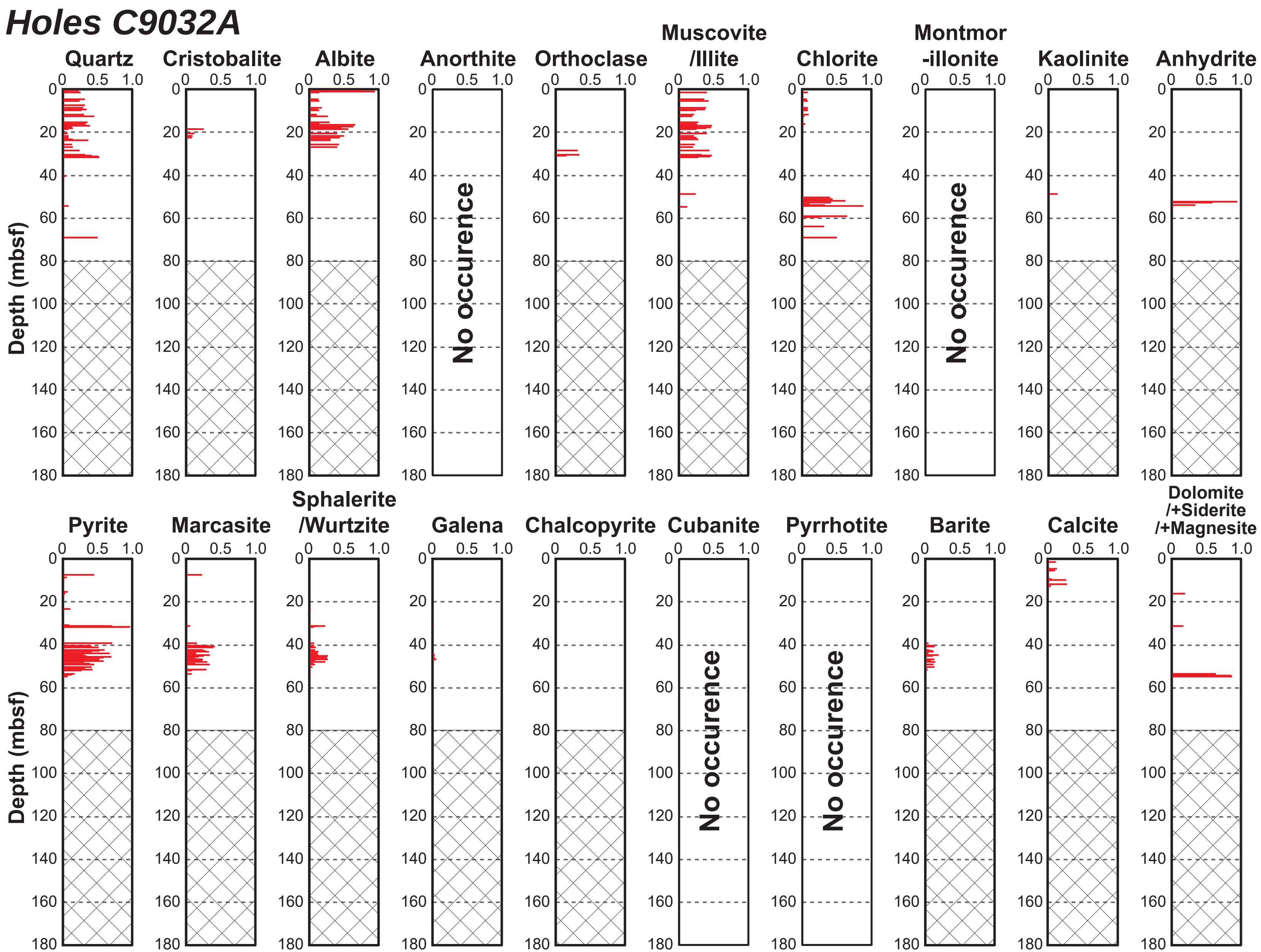

Supplementary Fig. S8 | Depth profile of constituent mineral fractions in Hole C9032A. Details are the same as in Supplementary Fig. S4.

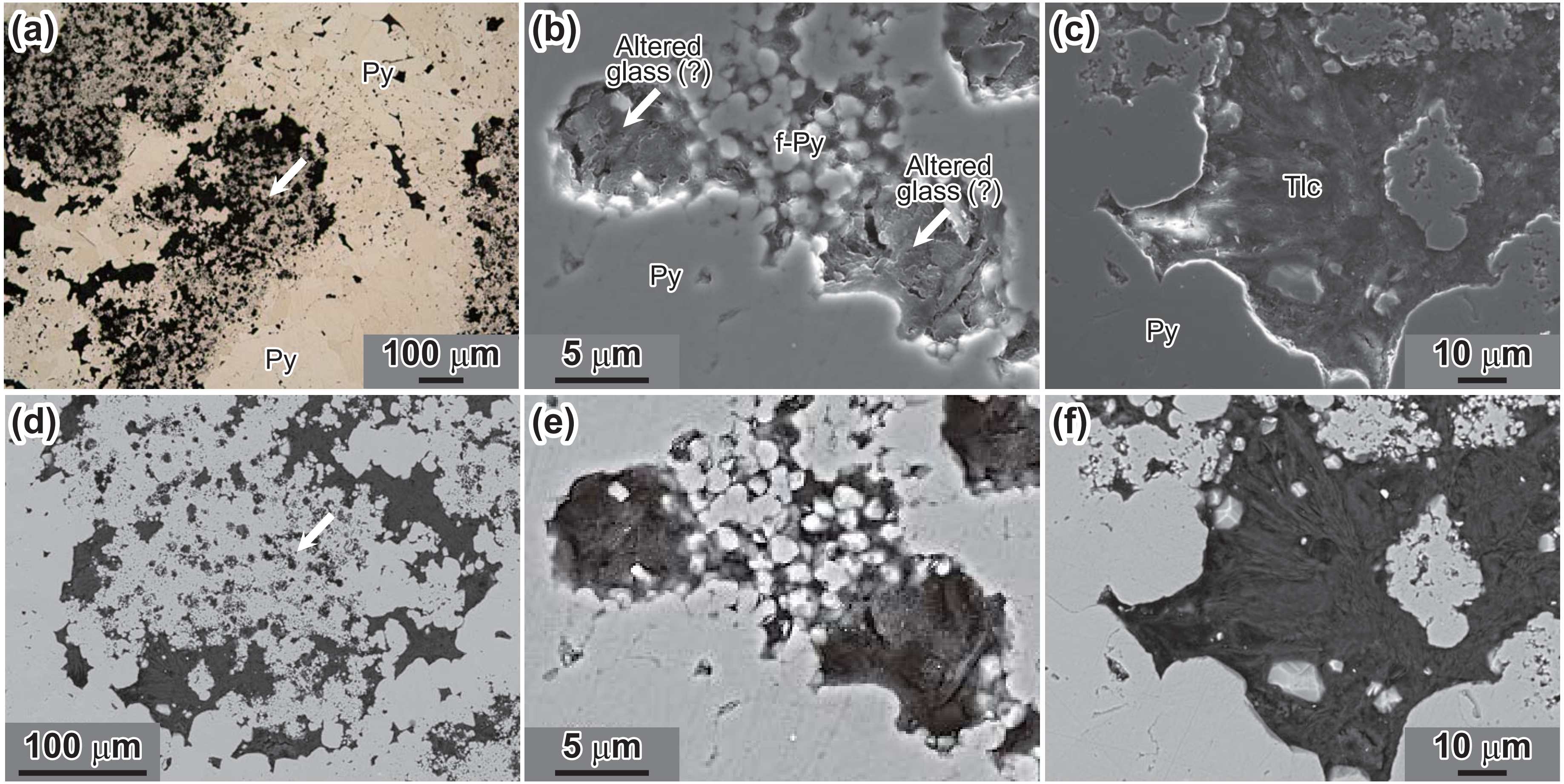

Supplementary Fig. S9 | (a) Microphotographs, (b, c) SEM images and (d–f) back-scattered electron images of sulphidic rock of the Northern Mound (Hole C9027B 8X-CC-W, 14.0–16.0 cm; 53.64–53.66 mbsf). (**a**, **b**, **d**, **e**) Framboidal pyrite (f-Py) is closely associated with amorphous hydrothermally altered material, interpreted as being derived from altered glass in pumice fragments. Images (**b**) and (**e**) are enlargements of the area indicated by the arrow in (**a**) and (**d**), respectively. (**c**, **f**) Interspaces in recrystallised framboidal pyrite and colloform pyrite (Py) are filled with talc (Tlc), which can be clearly distinguished from the amorphous material associated with initial framboidal pyrite.

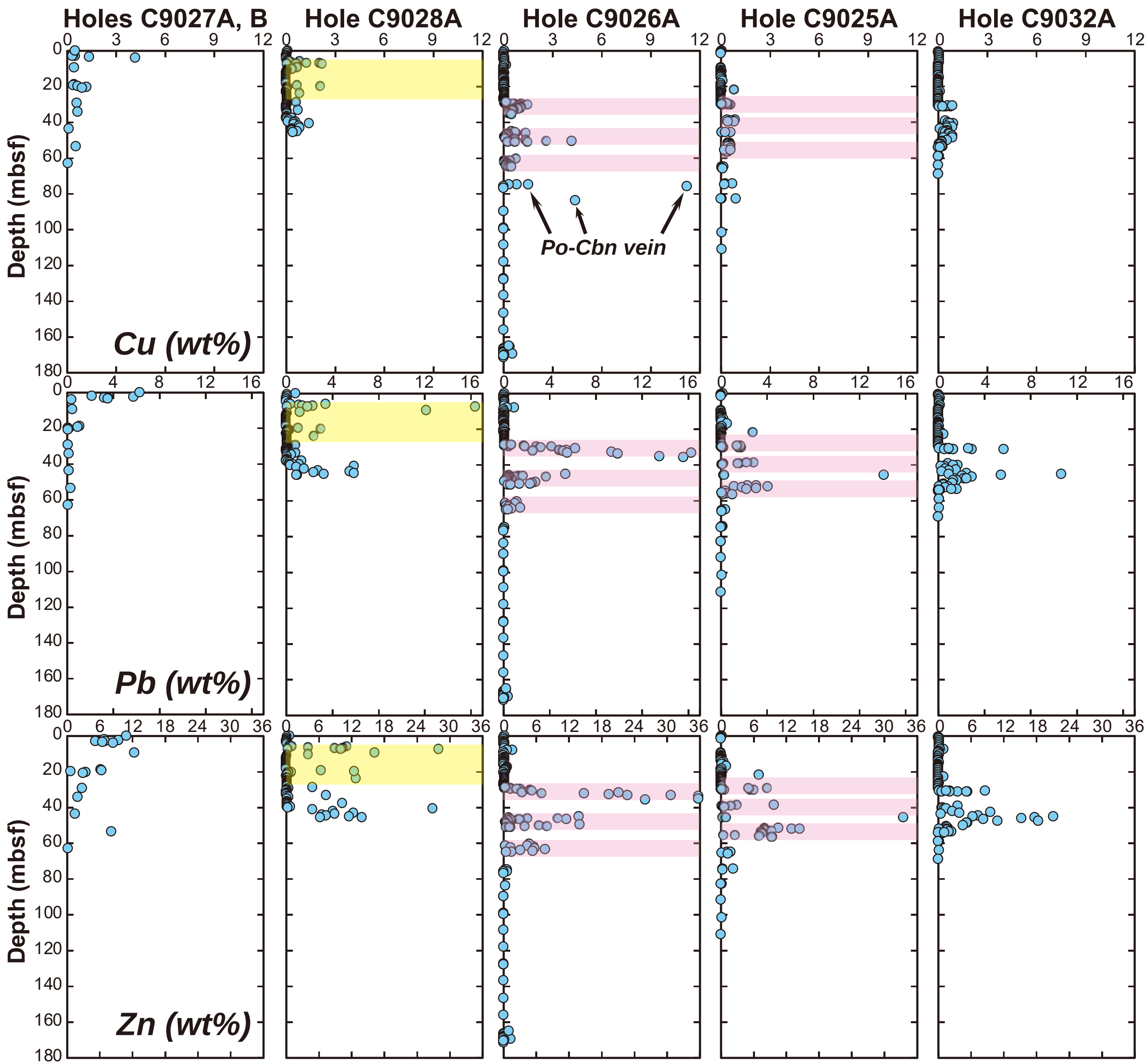

Supplementary Fig. S10 | Depth profiles of Cu, Pb and Zn concentrations in Holes C9027A/B, C9028A, C9026A, C9025A and C9032A from west to east determined by ICP-QMS. The subseafloor sulphide body in Holes C9026A and C9025A forms three layers (pink) separated by two hemipelagic sediment layers. The zone of highest Cu concentration below the sulphide body in Hole C9026A around 75 mbsf coincides with greenish chlorite-rich hydrothermally altered clay containing pyrrhotite-cubanite (isocubanite) (Po-Cbn) veins. Blocks enriched in Cu, Pb and Zn (shaded in yellow) above the sulphide body of Hole C9028A are derived from collapsed chimney and mound structures of the Northern Mound (Supplementary Figs. S1 and S2).

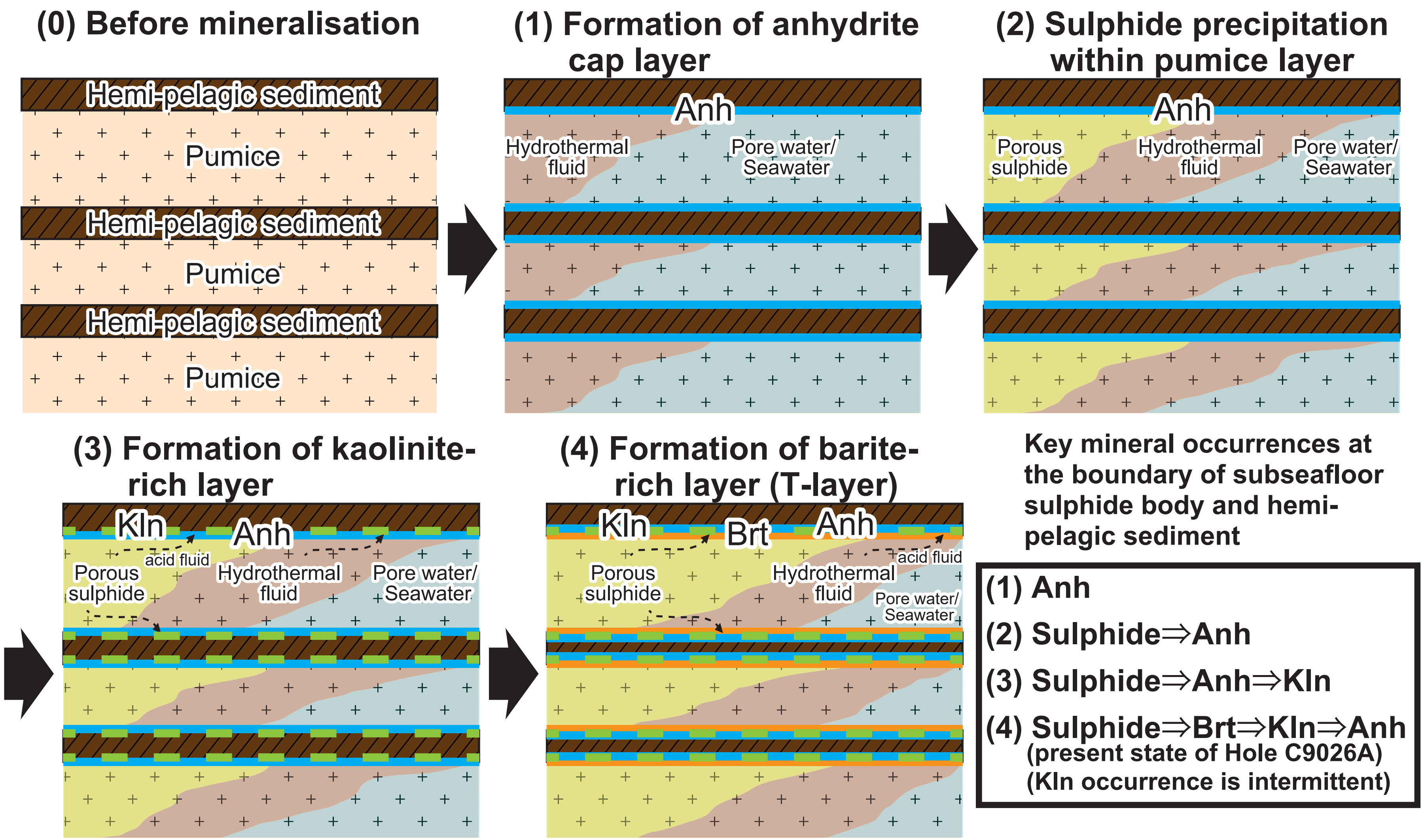

Supplementary Fig. S11 | Conceptual diagram of subseafloor sulphide mineralisation with pumice replacement. Alternating layers of permeable pumiceous material and hemipelagic sediment, the result of intermittent volcanism, function as a factory of subseafloor sulphide mineralisation (0). When hydrothermal fluid enters the pumice layer, anhydrite is precipitated as a cap layer (1). The temperature of the hydrothermal fluid beneath the cap becomes high enough to precipitate sulphide minerals (2). Kaolinite forms locally where the vapour phase of the hydrothermal fluid leaks through the cap (3). After hydrothermal activity ceases, anhydrite is replaced by barite and other sulphide minerals such that the key mineral occurrences across the T-layer (cap layer) are barite, kaolinite and anhydrite in ascending order (Fig. 2). Anh, anhydrite; Brt, barite; Kln, kaolinite.

References

44. Amante, C. & Eakins, B. W. ETOPO1 1 arc-minute global relief model: Procedures, data sources and analysis. NOAA Technical Memorandum NESDIS **NGDC-24**, https://doi.org/10.7289/V5C8276M (NOAA, 2009).

45. Kawada, Y. & Kasaya, T. Marine self-potential survey for exploring seafloor hydrothermal ore deposits. *Sci. Rep.* **7**, 13552 (2017).

46. Wessel, P. et al. The Generic Mapping Tools version 6. *Geochem. Geophys. Geosyst.* **20**, 5556–5564 (2019).
